# Assessing the diet and trophic level of marine fauna in a fishing ground subject to discarding activity using stable isotopes

**DOI:** 10.1371/journal.pone.0268758

**Published:** 2022-06-07

**Authors:** Benjamin Lejeune, Dorothée Kopp, Sonia Mehault, Maud Aline Mouchet

**Affiliations:** 1 Centre d’Ecologie et de Sciences de la Conservation, CNRS-MNHN-SU, Paris, France; 2 DECOD (Ecosystem Dynamics and Sustainability), IFREMER, INRAE, Institut Agro—Agrocampus Ouest, Lorient, France; University of Eldoret, KENYA

## Abstract

Discarding practices have become a source of concern for the perennation of marine resources, prompting efforts of discard reduction around the globe. However, little is known about the fate of discards in marine environments. Discarding may provide food for various marine consumers, potentially affecting food web structure and stability. Yet, quantifying reliance upon discards is difficult because identity and frequency of discards may change according to multiple factors, and most previously used diet assessment techniques do not allow to assume consistency of feeding strategies over time. One currently untested hypothesis is that significant contribution of discards over time should reflect in increased trophic level (TL) of marine fauna, particularly in low TL consumers. Here, we explored this hypothesis by modeling the TL and assimilated diet of consumers living in fishing grounds subject to important discarding activity using stable isotope analysis. We found indications that benthic invertebrates and Chondrichthyes may depict a higher than expected TL, while other fish tend to depict similar to lower TL compared to global averages from the literature. Based on prior knowledge of discard consumption in the same area, stable isotope mixing models congruently revealed that discards may represent substantial portions of the assimilated diet of most benthic invertebrate macrofauna, cephalopods and Chondrichthyes. We highlight limitations and challenges of currently used diet assessment techniques to study discard consumption and stress that understanding their reintegration in marine food webs is crucial in the context of an ecosystem approach to fisheries management and to better understand the functioning of marine ecosystems subject to fishing.

## Introduction

The trophic level (TL) of an organism expresses its position within the food web, with values set as one in primary producers and detritus, and increasing continuously from herbivores and detritivores up to higher level consumers [1, 2]. TL is an important concept informing about trophic interactions among organisms and defining their ecological role within food webs [2, 3]. Because trophic interactions are important drivers of ecosystem change [4], TL is often used as a key parameter to monitor and forecast impacts of anthropogenic changes on food webs and ecosystems (e.g. via calculation of trophic chain length, mean TL or bioaccumulation in food webs [3, 5, 6]).

Fishing activities can have complex cascading effects on marine food web structure and stability, potentially affecting ecosystem functioning [7, 8], and constitute one of the main global threats to marine ecosystems [9–11]. For example, fishing has been shown to globally impact the trophic level of marine fauna in overexploited fishing grounds by decreasing the mean TL of catches via the ‘fishing down marine food web’ effect [1, 12], while more specifically, bottom trawling has been shown to decrease the TL of some taxa by providing them with displaced small benthic fauna [13]. Recently, by-catches and subsequent discarding practices (i.e. animals caught, but returned to the sea, dead or alive, as a result of low commercial value, under quota restriction, below the minimum allowable size or damage), have been recognized as a source of concern for the perennation of marine resources prompting global efforts of discard reductions [14]. Discard rates are estimated to represent ~10% of global annual catches [14], but may vary considerably at lower spatial scale. Yet, little is known about the fate of discards in marine environments. While fisheries discards may benefit obligate scavengers, consumption by other facultative or non-typically scavenging species is considered to be certainly underestimated [9]. Multiple studies have shown that some benthic or demersal species may benefit from dead or damaged fauna linked to fishing as a source of food [15–17], but there is little information about their level of reliance upon discards on the long term, with large-scale model based ecosystem assessments providing contrasting results [18, 19]. One limitation is that empirical studies on discard consumption have relied mainly on underwater video recording [17, 20, 21] or traditional gut content analysis [22, 23], which only provide a snapshot of the diet and do not assume consistency of feeding strategies over time. Studying discard consumption is a timely issue since multiple countries recently adopted discard reduction policies (e.g. the European Landing Obligation; [24]) and a better understanding of discards reintegration in marine food webs may help forecast their potential consequences in ecosystems subject to fishing [9].

One may argue that the long-term nature of discarding practice and its importance in terms of biomass may have played an important role in shaping marine communities and food web structure in some fishing areas [14, 25]. Because the effect of discarding activity on marine communities may be difficult to observe or quantify, monitoring key parameters such as TL of consumers may inform about the importance of discard consumption across a community. One currently untested hypothesis is that if discard contribution to the diet of marine fauna is important over time, it should reflect in an increased TL, as hypothesized based on the studies documenting effects of trawling on the TL of benthic consumers low in the food web [26, 27]. Recent developments in Bayesian stable isotope mixing models allow modeling of TL and reconstructing assimilated diet based on the isotopic composition of consumers and their prey, while propagating uncertainties linked to isotopic variability in the models [28, 29]. Stable isotopes are trophic tracers that have been widely used to depict the assimilated diet of consumers in a time- and space-integrative manner and its variation according to biological or environmental factors, notably in marine environments [30–32]. Two elements, nitrogen (N) and carbon (C), are most commonly employed as they allow describing both horizontal and vertical dimensions of food webs [33]. Ratio of carbon ^13^C/^12^C isotopes of a consumer (δ^13^C, expressed relative to an international standard) can inform about its basal sources of dietary carbon as δ^13^C changes little with trophic transfers but varies substantially among primary producers depicting different photosynthetic pathways [34, 35]. Ratio of nitrogen ^15^N/^14^N isotopes of a consumer (δ^15^N) exhibits stepwise enrichment with trophic transfers, and can therefore be used to estimate its TL over a period of time corresponding to the turn-over rate of the sampled tissue [2]. Stable isotopes would constitute a useful complementary tool to assess the fate of discards in marine environments by measuring their contribution to the assimilated diet of marine consumers (as a complement to knowledge of the ingested diet obtained via traditional gut content analysis) and integrating diet information over a longer period of time than traditionally used techniques. Identifying taxa that may benefit from discards is an important issue, since current efforts to limit discarding practices (e.g. European Landing Obligation; [24]), may differently affect marine consumers in fishing areas and potentially propagate unanticipated changes through local food webs [16, 36–38].

Here, we aimed to depict trophic level and food web structure of consumers feeding in a coastal fishing ground subject to important discarding activity (i.e. the Bay of Bourgneuf, northeast Atlantic, France) a year before the full implementation of the European Landing Obligation (i.e. set to be fully implemented in 2020 in the area [39, 40]). Discard data collected in the area of study suggest that the application of the Landing Obligation should significantly reduce discarding by trawlers in the Bay of Bourgneuf [38, 39] and potentially affect a wide diversity of bentho-demersal species suspected to rely on discards based on gut content analysis using DNA metabarcoding [38]. However, whether discard ingestion identified by gut content analysis correspond to long-term feeding strategies remains to be established. To this end, we further looked for (1) indications of TL variation compared to global mean TL from the literature and (2) evidence of fisheries discards assimilation of selected consumers based on prior knowledge of discard ingestion [38]. We modeled the trophic level and assimilated diet of marine consumers feeding in fishing grounds of the Bay of Bourgneuf using carbon and nitrogen stable isotope mixing models in a Bayesian framework.

## Material and methods

### Sampling and stable isotope data acquisition

**Fig 1:**
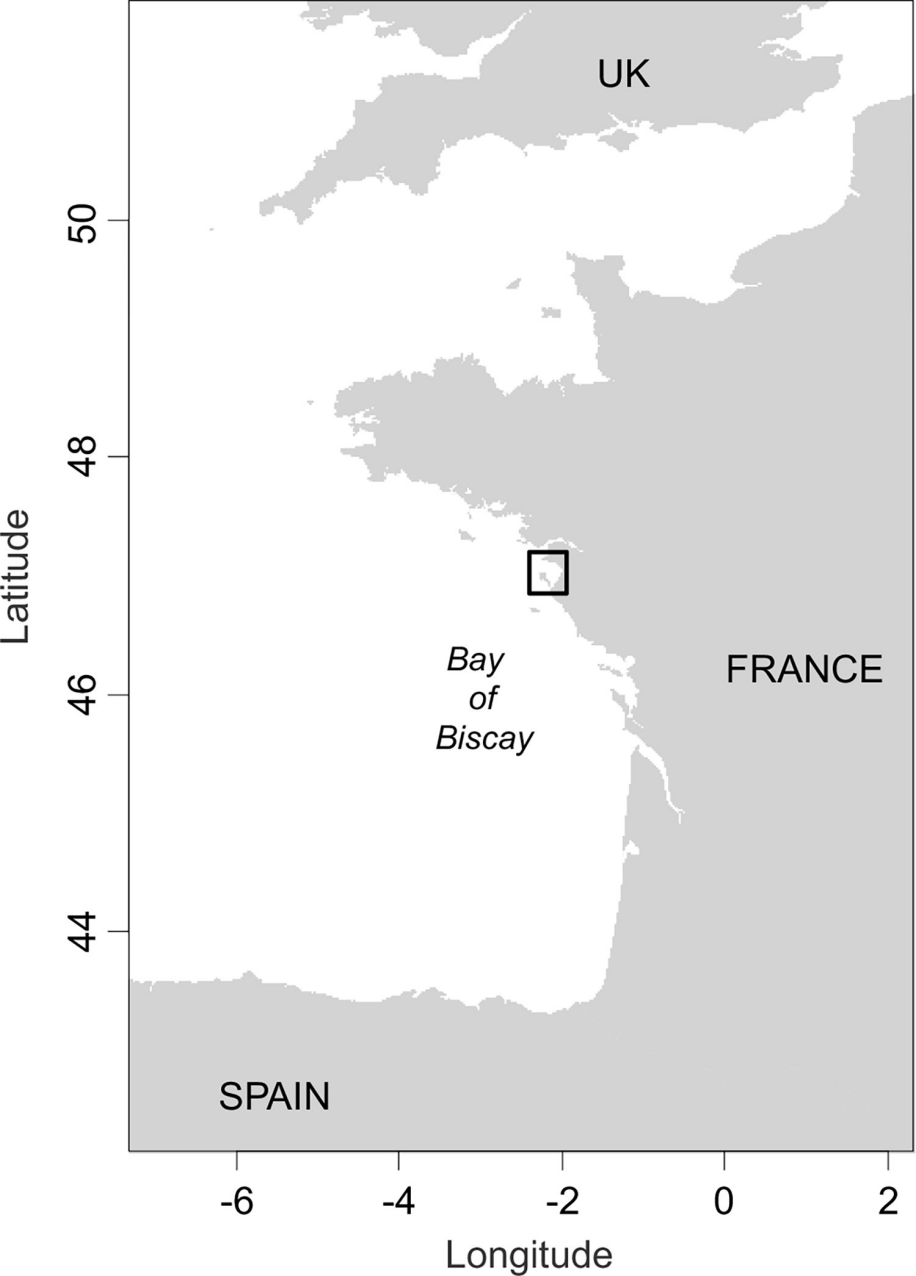
Map of the sampling location. The Bay of Bourgneuf, a shallow bay (from 0 to 34m depth) covering a relatively small area (320 km^2^) is located within the Bay of Biscay (France, northeast Atlantic).

Sampling was conducted in April 2019 in the Bay of Bourgneuf (Bay of Biscay, France, northeast Atlantic) (Fig 1). It is a shallow bay (from 0 to 34m depth), covering a relatively small area (320 km^2^), diverse both in terms of substrate type (i.e. composed of a variety of patchy rocky, sandy and muddy bottoms) and species occurrence [41]. Fishing and discarding activities occur all year round in the Bay of Bourgneuf [36, 42]. Sampling was carried out between 0 and 22 m depth, within an area of commercial fishing and on board a 10.95 m long commercial trawler rigged with a single bottom trawl targeting multispecies fish assemblages (20 m headline and 70 mm diamond mesh codend). Individuals were randomly selected from each trawl to be sampled for stable isotope analysis. In total, stable isotope analyses were performed on 216 individuals belonging to 31 consumer taxa (*n* = 4 to 9 individuals), spanning 7 different taxonomic classes (18 Actinopterygii, 2 Chondrichthyes, 2 Cephalopoda, 6 Decapoda, 1 Gasteropoda, 1 Polychaeta) and 1 Bivalvia. Captured individuals were dissected and directly frozen at −20°C on board. Samples consisted of dorsal muscle for fish, muscle from the mantle for cephalopods (*Alloteuthis spp*. and *Sepia officinalis*), feet muscle for gasteropods (*Buccinum undatum*), adductor muscle for bivalves (*Pecten maximus*), leg muscles for crabs (*Atelecyclus undecimdentatus*, *Cancer pagurus* and *Necora puber*), hermit crabs (*Pagurus spp*.) and spider crabs (*Maja brachydactyla*), abdominal muscle for shrimps (*Crangon spp*.) and a piece of the body excluding digestive tract for polychaetes (*Aphrodita aculeata*). Samples were stored frozen then freeze-dried and ground to a fine powder in the laboratory. Determination of δ^15^N, δ^13^C and % content of C and N was done at the Cornell University Stable Isotope Laboratory. Analyses were performed on a Thermo Scientific Delta V isotope ratio mass spectrometer (IRMS) interfaced to a NC2500 elemental analyzer. Stable isotope ratios were expressed following the classical δ notation, as deviation from international standards (Vienna Pee Dee Belemnite for δ^13^C and atmospheric N_2_ for δ^15^N). Data were normalized and checked using internal lab standards (corn, trout, and deer) and instrument linearity was measured using a chemical standard (Methionine). Standard deviation on repeated measures was < 0.15 for δ^13^C and < 0.2 for δ^15^N. Samples were not acidified since calcified structures were mechanically removed during dissection and preparation (e.g. mollusk shell, decapod cuticle) and no lipid correction was applied since all samples had a C/N ratio < 4 (i.e. proxy for lipid content [43]). All data were collected and analyzed in accordance with the authorization obtained by written consent and delivered by the Direction interrégionale de la mer Nord Atlantique–Manche Ouest (decision: 39/2019).

### Trophic level modeling using stable isotopes

We estimated the trophic level (TL) of each consumer using a two baselines (benthic-pelagic) Bayesian mixing model in the tRophicPosition R package [29] in R version 4.0.5 [44]. This approach does not require making any assumptions about the respective importance of baselines for TL calculation, which may be more suitable in shallow coastal areas where benthic-pelagic coupling is generally stronger [45]. The Bayesian framework allows propagating uncertainties linked to variability in trophic discrimination factors (TDFs), sources and consumer isotopic compositions. For Actinopterygii and Chondrichthyes, TDFs were set to 1 ± 0.5‰ for carbon [45]. For nitrogen, we used a TDF of 3.4 ± 0.5‰ for Actinopterygii and 2.3 ± 0.5‰ for Chondrichthyes following [45, 46], so as to cope with metabolic differences in N assimilation between these two classes. For all invertebrates, we used TDFs of 0.5 ± 0.13‰ and 2.3 ± 0.18‰ for carbon and nitrogen, respectively [47]. Scallops (*Pecten maximus*, TL = 2 [48]) were used as the benthic baseline in the models following [45]. Because the package requires that both benthic and pelagic baselines are of equal TL, we retrocalculated the isotopic composition of the pelagic primary consumer (PPC) from that of Sprat (*Sprattus sprattus*), using the simulateTDF function of tRophicPosition (*n* = 50, with a random resampling of 7) and the previously detailed TDFs for Actinopterygii. *S*. *sprattus* is a strictly zooplanktivorous species [49] and the most pelagic species in the sampled community according to its isotopic composition. Model parameters were: lambda = 2, chains = 3, adaptive iterations = 10,000, iterations = 500,000, burn-in = 100,000 and thin = 100. We compared the modeled TL with global averaged TL from the literature (mean ± SE) retrieved from www.fishbase.org [50] and www.sealifebase.org [51] databases, for fish and invertebrate taxa respectively to obtain insights into potential variation linked to discard consumption. TL which were not available in these databases were retrieved from www.seaaroundus.org/ [52].

### Diet reconstruction using stable isotope mixing models

We further explored the assimilated diet of invertebrate macrofauna and Chondrichthyes based on prior evidence of discard ingestions from stomach content data collected on the same field campaign (unpublished data) that were used as a basis for source selection in Bayesian mixing models computed in ‘MixSIAR’ R package [28]. Because having too many sources relative to the number of tracers reduces the discriminatory ability of mixing models [53], potential food sources were grouped *a priori* using a hierarchical clustering approach. This approach allowed us to depict the consumer food web structure. To avoid biases in within-sample isotopic variation linked to different sample sizes among taxa in our dataset, we performed UPGMA (unweighted pair group method with arithmetic mean [54]) clustering on a bootstrapped matrix of distances between taxa, as follow: We sampled 4 individuals with replacement per taxon (i.e. minimum sample size in our dataset) and used the isotopic ratios of these samples as coordinates to compute a Euclidian distance matrix between taxa, after standardizing coordinates to 0 mean and unit variance. We repeated this procedure 500 times (a sufficient number to stabilize the values of the bootstrapped distance matrix) and the bootstrapped distance matrix was obtained by averaging the resulting 500 distance matrices. UPGMA clustering was performed using the ‘cluster’ R package [55]. We assessed the optimal number of clusters to consider for mixing model analysis by visual inspection of the resulting clustering dendrogram, further confirmed using graphs of the fusion level [56].

For each consumer taxa, we selected potential food sources to be implemented in mixing models among the trophic guilds obtained via clustering, based on prior diet knowledge of each consumer from the literature and local stomach content records (unpublished data). We subsequently pooled different guilds together to further reduce the number of different food sources in the models whenever it would improve model performances. Mixing models were set to account for process and residual errors and minimum MCMC parameters were: 3 chains, length = 100,000, burn-in = 50,000 and thin = 50. We used the same TDFs as for TL modelling, as described above. Diet reconstruction using mixing models were performed only on consumer taxa which fitted well within the polygon of sources, excluding consumers for which some important food sources were not collected according to their diet in the literature. Excluded taxa were: *Crangon spp*. and *Pagurus spp*. Markov Chain convergence was assessed by visual inspection of trace plots, complemented with Gelman-Rubin, Geweke, and Heidelberger and Welch diagnostics. We used Deviance Information Criterion (DIC) to compare model performances and select those that were most supported by the data [57]. Only the most performant model for each consumer was presented but different ways of pooling sources did not critically alter the interpretation of model solutions.

## Results

### Trophic level modeling

*Callionymus lyra*, *Scomber scombrus* and Clupeidae (*Engraulis encrasicolus*, *Sardina pilchardus*, *Sprattus sprattus*) occupied the lowest trophic levels (TL), with TL mean and 95% credible intervals being respectively 2.8 (2.6–3.1), 2.8 (2.5–3.2), 3.1 (2.8–3.4), 3.0 (2.7–3.4), 2.9 (2.7–3.1) corresponding to secondary consumers (Table 1). The highest TL corresponded to tertiary consumers (TL > 4) and were occupied by the cephalopods *Alloteuthis spp*. 4.4 (3.7–5.2) and *Sepia officinalis* 4.2 (3.7–4.8), and the demersal and benthic fishes *Merlangius merlangus* 4.2 (3.9–4.5), *Scyliorhinus canicula* 4.3 (3.9–4.7) and *Raja undulata* 4.1 (3.8–4.5). Decapods occupied trophic levels corresponding to secondary-tertiary consumers, ranging between 3.2 (2.9–3.6) for the circular crab *Atelecyclus undecimdentatus* and 3.8 (3.5–4.4) for *Crangon spp*; almost identical to *Cancer pagurus* (TL = 3.8 [3.4–4.4]). The gastropod *Buccinum undatum* and the polychaete *Aphrodita aculeata* occupied similar TL: 3.7 (3.3–4.4) and 3.8 (3.3–4.5), respectively.

**Table 1:** Comparison between global averaged trophic level (TL) and TL modeled from isotopic composition of the 30 consumers. Global averaged TL are presented as mean ± SE and derived from fishbase and sealifebase databases, except for *Cancer pagurus* and *Necora puber* which are derived from seaaroundus database. Modeled TL are provided as mean and 95% credible intervals (CI) and calculated using tRophicPosition R package. ‘n’ = sample size. ‘Code’ = simplified taxa names used in figures. ‘Cluster’ = trophic group according to UPGMA clustering.

					Stable isotopes		Global averaged TL		Modeled TL	
Class	Taxon	n	Code	Cluster	δ^13^C (‰)	δ^15^N (‰)	Mean	SE	Mean	CI
Actinopterygii	*Callionymus lyra*	7	Clyr	BIF	−17.4 ± 0.2	12.6 ± 0.6	3.3*	0.4	2.8	2.6–3.1
Actinopterygii	*Chelidonichthys lucerna*	7	Cluc	SBF	−15.7 ± 0.9	15.3 ± 0.3	4	0.1	3.6	3.4–3.8
Actinopterygii	*Conger conger*	6	Ccon	DFC	−16.8 ± 0.5	15.3 ± 0.7	4.3	0.4	3.6	3.3–4.0
Actinopterygii	*Engraulis encrasicolus*	7	Eenc	PSC	−18.2 ± 0.2	13 ± 0.6	3.1*	0.4	3.1	2.8–3.4
Actinopterygii	*Sardina pilchardus*	6	Spil	PSC	−18.3 ± 0.6	12.8 ± 0.7	3.1	0.1	3.0	2.7–3.4
Actinopterygii	*Sprattus sprattus*	7	Sspr	PSC	−19.3 ± 0.3	12 ± 0.5	3*	0.1	2.9	2.7–3.1
Actinopterygii	*Scomber scombrus*	7	Ssco	PSC	−18.5 ± 0.5	12.1 ± 0.8	3.6*	0.2	2.8	2.5–3.2
Actinopterygii	*Osmerus eperlanus*	7	Oepe	DFC	−17.4 ± 0.6	15.5 ± 0.5	3.5*	0.4	3.8	3.6–4.1
Actinopterygii	*Belone belone*	4	Bbel	BIF	−17.4 ± 0.6	13.4 ± 1.5	4.2	0.4	3.2	2.5–4.2
Actinopterygii	*Trachurus trachurus*	7	Ttra	DFC	−18.1 ± 0.4	15 ± 0.6	3.7	0.0	3.8	3.5–4.0
Actinopterygii	*Pollachius pollachius*	7	Ppol	DFC	−17.2 ± 1	15.2 ± 0.6	4.3	0.3	3.7	3.4–4.0
Actinopterygii	*Trisopterus luscus*	7	Tlus	DFC	−16.7 ± 0.7	15.7 ± 0.9	3.7	0.1	3.8	3.4–4.2
Actinopterygii	*Merlangius merlangus*	6	Mmerla	DFC	−17.5 ± 0.3	16.7 ± 0.6	4.4	0.2	4.2	3.9–4.5
Actinopterygii	*Merluccius merluccius*	7	Mmerlu	DFC	−18 ± 0.4	14.5 ± 0.7	4.4	0.0	3.6	3.3–3.9
Actinopterygii	*Pagrus pagrus*	7	Ppag	DFC	−16.6 ± 0.5	15.8 ± 0.5	3.9	0.2	3.8	3.5–4.0
Actinopterygii	*Spondyliosoma cantharus*	7	Scant	DFC	−17 ± 0.6	15.3 ± 0.5	3.3	0.2	3.7	3.4–4.0
Actinopterygii	*Labrus bergylta*	4	Lber	BIF	−17.4 ± 1	13.8 ± 0.6	3.2	0.0	3.3	2.8–3.8
Actinopterygii	*Solea solea*	7	Ssol	BIF	−17.2 ± 1.4	14.1 ± 0.4	3.2	0.2	3.3	3.1–3.6
Chondrichthyes	*Raja undulata*	7	Rund	SBF	−16 ± 0.6	14.6 ± 0.4	3.5*	0.4	4.1	3.8–4.5
Chondrichthyes	*Scyliorhinus canicula*	7	Scani	DFC	−16.8 ± 0.3	14.6 ± 0.4	3.8	0.3	4.3	3.9–4.7
Cephalopoda	*Alloteuthis spp*.	7	Allo	DFC	−18.6 ± 0.5	14.6 ± 1.3	3.5*	0.4	4.4	3.7–5.2
Cephalopoda	*Sepia officinalis*	7	Soff	DFC	−17.3 ± 0.2	14.7 ± 0.4	4.3	0.7	4.2	3.7–4.8
Decapoda	*Atelecyclus undecimdentatus*	9	Aund	BIF	−17.1 ± 0.8	12.5 ± 0.5	> 2.8	NA	3.2	2.9–3.6
Decapoda	*Cancer pagurus*	7	Cpag	BIF	−17.3 ± 0.5	13.9 ± 0.7	3.1	NA	3.8	3.4–4.4
Decapoda	*Necora puber*	7	Npub	BIF	−16.9 ± 0.3	12.9 ± 0.6	2.6	NA	3.5	3.1–4.0
Decapoda	*Maja brachydactyla*	7	Mbra	BIF	−16.8 ± 0.4	13.3 ± 0.7	3.2*	0.2	3.6	3.2–4.2
Decapoda	*Pagurus spp*.	7	Pagu	BIF	−16.2 ± 0.5	12.9 ± 0.3	3.6*	0.2	3.4	3.1–3.9
Decapoda	*Crangon spp*.	7	Cran	SBF	−15.3 ± 1.2	13.9 ± 0.2	3.2	0.5	3.8	3.5–4.4
Gasteropoda	*Buccinum undatum*	7	Bund	BIF	−16.4 ± 0.5	13.4 ± 0.7	3.4*	0.4	3.7	3.3–4.4
Polychaeta	*Aphrodita aculeata*	6	Aacu	BIF	−16.6 ± 0.7	13.7 ± 0.7	3.2	0.4	3.8	3.3–4.5

* = Mean and SE calculated via randomized resampling of diet items when diet proportions were not available [50, 51].

Chondrichthyes, squids *Alloteuthis spp*. and most benthic invertebrates depicted relatively high TL compared to global averaged TL, but standard errors of those global averages were generally overlapping with the lower end of the 95% CI of TL modeled from consumers isotopic composition. In other fish, modeled TL were either very close or lower than mean TL from the literature, but again mostly overlapping with standard errors of those estimates when considering the 95% CI around the mean of modeled TL. Variability in TL posterior estimates (95% CI around the mean) was generally large in invertebrates, ranging between 0.7 and 1.5 TL for *A*. *undecimdentatus* and *Alloteuthis spp*., and lower in fish (0.4–0.8 TL ranges), except for *Labrus bergylta* and *Belone belone* (1 and 1.7 TL ranges, respectively).

**Fig 2:**
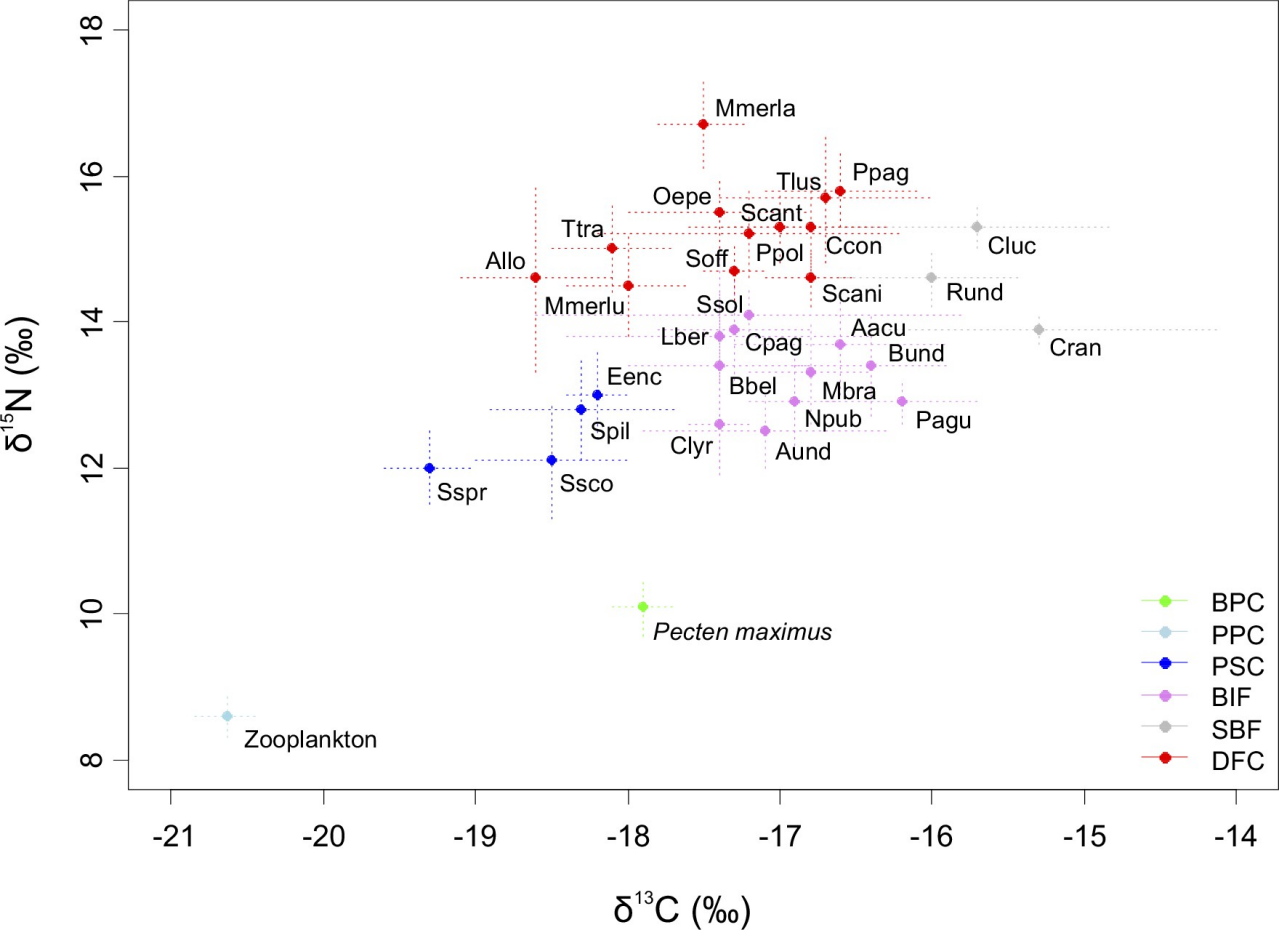
Stable isotope composition of the species sampled in the Bay of Bourgneuf. Data is presented as mean ± SD and species are organized in 6 different trophic groups according to hierarchical clustering. BPC = Benthic primary consumers (green). PPC = Pelagic primary consumers (light blue). PSC = Pelagic secondary consumers (blue). BIF = Benthic invertebrates and fish (black). SBF = Shrimps and benthic fish (grey). DFC = Demersal fish and cephalopods (red). Consumers’ abbreviations are provided in Table 1.

Hierarchical clustering performed on δ^13^C and δ^15^N values of sampled marine fauna revealed six different trophic groups (Fig 2; S1 Fig). Two groups of primary consumers could be distinguished: Benthic primary consumers (BPC), represented by the scallop *Pecten maximus* (δ^13^C = −17.9 ± 0.2; δ^15^N = 10.1 ± 0.4), and pelagic primary consumers (PPC), composed of zooplankton (δ^13^C = −20.4 ± 0.2; δ^15^N = 8.6 ± 0.3; values retro-calculated from Sprat, see details in Material and Methods). One group of pelagic secondary consumers (PSC; δ^13^C = −18.6 ± 0.6; δ^15^N = 12.5 ± 0.8), composed of the three Clupeidae species and *Scomber scombrus*, could be distinguished (Table 1). One cluster regrouped higher TL demersal fishes and cephalopods (DFC; δ^13^C = −17.3 ± 0.8; δ^15^N = 15.2 ± 0.9). Finally, two groups composed of both benthic invertebrate macrofauna and fish could be distinguished. Benthic invertebrates and fishes (BIF; δ^13^C = −16.9 ± 0.7; δ^15^N = 13.2 ± 0.8) regrouped all benthic invertebrate taxa excluding *Crangon* shrimps and four low TL fishes (*Belone belone*, *Callionymus lyra*, *Larbus bergylta* and *Solea solea*). Shrimps and benthic fishes (SBF; δ^13^C = −15.7 ± 0.9; δ^15^N = 14.6 ± 0.7) regrouped *Crangon* shrimps, *Chelidonychthys lucerna* and *Raja undulata*.

### Potential contribution of fisheries discards to consumers’ diet

Stable isotope mixing models revealed that ‘benthic primary consumers’ (BPC) was the main food source for all Chondrichthyes and invertebrate macrofauna, except for *Alloteuthis spp*. Consumption of this food source ranged from mean = 39% (95% CI = 18–56) to 87% (70–96) for *Scyliorhinus canicula* and *Atelecyclus undecimdentatus*, respectively (Table 2). While they relied similarly on benthic primary consumers, the diet of the two Chondrichthyes clearly differed according to their consumption of other food sources. *Raja undulata* relied more on trophic groups that mainly included benthic secondary consumers (SBF–BIF = 30% [3–54]), while *Scyliorhinus canicula* relied more on pelagic secondary consumers (PSC = 28% [4–55]) and high TL demersal species (DFC = 20% [2–39]). Yet, *R*. *undulata* still preyed upon pelagic secondary consumers and demersal fish and cephalopods, both representing 12–14% mean contributions to its diet. Pelagic primary and secondary consumers made up most of *Alloteuthis spp*. diet (PPC–PSC = 72% [53–89]), but with a substantial contribution of prey from high TL demersal prey (DFC = 19% [2–39]), while benthic fish and invertebrates represented less important food sources (SBF–BIF = 7% [0–28]). By contrast, *S*. *officinalis* relied mainly on benthic primary consumers (41% [19–57]), followed by even contributions of pelagic secondary consumers and higher TL demersal prey (24% [3–41] and 25% [4–52]) and similarly lower contributions of other benthic prey. Among decapods and congruent with trophic level modelling, *Atelecyclus undecimdentatus* depicted a diet almost exclusively based on benthic primary consumers, while *Cancer pagurus* was the least reliant upon this source (56% [32–74]), and the most reliant upon pelagic and demersal fish and cephalopods (PSC = 18% [1–47] and DFC = 12% [1–30]) with 11% (1–30) contribution of other benthic prey. Other benthic invertebrate taxa (decapods, polychaetes and gastropods) depicted a relatively similar diet whereas benthic primary consumers constituted between 57 and 74% (mean) of assimilated prey, followed by prey mainly including benthic secondary consumers (i.e. SBF–BIF representing mean contributions between 15 and 25%) and lower contributions of pelagic and demersal fish and cephalopods (mean contributions between 4 and 8% each).

**Table 2:** Stable isotope mixing models depicting the assimilated diet of 10 taxa with prior knowledge of important discard ingestion in the Bay of Biscay (France). Food sources were combined in 6 groups *a priori* using a hierarchical clustering approach: BPC = Benthic primary consumers, SBF = Shrimps and benthic fish, BIF = Benthic invertebrates and fish, DFC = Demersal fish and cephalopods, PPC = Pelagic primary consumers and PSC = Pelagic secondary consumers. Contributions are presented as mode and 95% credible intervals of diet proportions. NA = source not included in the model. See Material and methods and Table 1 for the list of taxa included within each food source group.

Consumer	BPC	SBF—BIF	DFC	PSC	PPC
*R*. *undulata*	42% (21–57)	30% (3–54)	14% (1–38)	12% (1–43)	NA
*S*. *canicula*	39% (18–56)	13% (1–32)	20% (2–39)	28% (4–55)	NA
*Alloteuthis spp*.	NA	7% (0–28)	19% (2–39)	72% (53–89)*	
*S*. *officinalis*	41% (19–57)	10% (1–28)	24% (3–41)	25% (4–52)	NA
*A*. *undecimdentatus*	87% (70–96)	4% (0–17)	3% (0–11)	4% (0–19)	NA
*C*. *pagurus*	56% (32–74)	11% (1–30)	12% (1–30)	18% (1–47)	NA
*N*. *puber*	74% (54–88)	15% (1–33)	5% (0–19)	4% (0–21)	NA
*M*. *brachydactyla*	67% (48–83)	18% (2–35)	6% (0–24)	6% (0–27)	NA
*A*. *aculeata*	57% (31–75)	24% (3–44)	8% (0–29)	8% (0–39)	NA
*B*. *undatum*	63% (41–78)	25% (5–45)	5% (0–23)	5% (0–25)	NA

* = PSC and PPC sources were pooled together in *Alloteuthis spp*. mixing models. SBF and BIF sources were pooled in all models.

## Discussion

### Discard consumption and its potential effect on trophic level

Previous studies have suggested that discarding practices may enhance secondary production in marine food webs, notably by providing an abundant food source for a variety of demersal and benthic consumers [19, 20, 25]. If this is true and subsidies are stable, then one may expect benthic and demersal discard consumers to depict a higher trophic level (TL) in areas subject to important discarding, particularly in low TL species. Posterior distributions of modeled TL were sometimes relatively spread, leading to large 95% credible intervals around the mean. Given the underlying consumers isotopic variability (Table 1), this may correspond to trophic variability related to mixed, generalist or opportunistic feeding behaviors which characterize most of these species and is also congruent with isotopic variability in other studies [45, 50, 51]. Except for the garfish *Belone belone*, TL ranges were generally larger in invertebrates (95% CI close to 1 or higher, and highest in *Alloteuthis spp*.: 95% CI range = 1.5 TL) compared to fish, potentially indicating a higher degree of omnivory (i.e. feeding at multiple trophic levels). Comparison of local modeled TL with global averages available from fishbase [50] and sealifebase [51] or seaaroundus [52] databases only provide indications and do not constitute formal tests. Indeed, TL may be influenced by many environmental factors including prey availability and habitat structure. Therefore, potential geographic or environmental discrepancies between our samples and those underlying global averages may bring uncertainty in such comparisons. Additionally, variability in global TL estimates is expressed as standard error which is not always calculated following the same procedure depending on the underlying available data [50, 51] or were not available for certain taxa. Different body size distributions between populations used to calculate global averaged TL from the literature and modelled TL could also potentially introduce a bias in their comparison as body size is an important determinant of the trophic ecology [58]. However, both simulation and empirical data support the existence of negative TL—body size relationships in marine food webs, suggesting that body size may not always be predictive of the TL in marine species [59]. The limited sample size within each consumer species did not allow us to test for the effect of body size on the diet or TL of sampled consumers, but individuals generally displayed little variation in body size within each species (see S1 Table), which should therefore not be responsible for large CI observed in the TL of some species. In addition, we limited this potential source of bias by selecting global averaged TL calculated on the same ontogenetic stages or size class as our sampled populations, whenever available. Despite these limitations, modeled TL suggest an opposite pattern whereas most sampled invertebrates (decapods, polychaetes and squids) and both Chondrichthyes depicted TL that tended to be higher than expected according to global averages while other fish depicted TL that tended to be either similar or lower than expected [50, 51]. Global averages may still be potentially influenced by discarding, but these are often used to assign theoretical TL to species in ecosystem models and the studied area is expected to be much more influenced due to its specificities (e.g. shallow, semi-enclosed, year round fishing and discarding). The observed pattern echoes with previous research linking trawling to increased δ^15^N values of some benthic or demersal species, potentially influenced by feeding on dead fauna originating from trawling [13, 26, 60]. Higher than expected TL in decapods, polychaetes and squids are congruent with gut content metabarcoding results revealing that these taxa may be among the main discard consumers in the Bay of Bourgneuf [38]. Complex and contrasting effects suggest that in trawled areas both discard consumption (generally higher TL fauna) and the consumption of dead, damaged or displaced small benthic fauna due to seabed perturbation (generally lower TL fauna) may affect the δ^15^N of demersal species in opposite ways (i.e. increasing or decreasing it, respectively) [60]. For example, in the Celtic sea, whiting (*Merlangius merlangus*) and large body size megrim sole showed significantly higher δ^15^N values at low trawling effort sites, while there was no effect at higher trawling pressure [13]. In light of these results, one may argue that at low trawling pressure, discarding may increase the TL of some fish such as gadoids or flatfishes which are known to scavenge upon discards [15, 16], but at higher trawling pressure, the increased availability of low TL benthic prey may become more profitable or counterbalance any visible effect of discard consumption on their TL.

Previously observed patterns of δ^15^N values decrease with trawling intensity in piscivorous fish [13, 60, 61] might corroborate with some of our results, showing that several fish depicted TL that tended to be lower than expected (i.e. pelagic species *Belone belone* and *Scomber scombrus*, and bentho-demersal species *Callionymus lyra*, *Conger conger*, *Merluccius merluccius* and *Pollachius pollachius*). It is possible that most fish in our study take advantage of an increase in low TL benthic fauna availability due to trawling activity, or naturally, in accordance with the increased benthic-pelagic coupling revealed by stable isotope analysis in low-depth coastal areas [45, 62]. This could counter balance any potential effect of discard consumption (identified in many of these species in the Bay of Bourgneuf using gut content metabarcoding [38]) on their TL. This would also be consistent with previous studies showing that gadoids and many perciformes such as Callionymidae, Scombridae or Sparidae are frequently observed to scavenge upon both discards and carrion generated by trawling [15, 18, 63], but remains at this step speculative. Alternatively, scavenging behaviors revealed in other studies may not correspond to consistent long-term strategies, which would therefore not reflect in their isotopic composition. Other factors at play might be that for piscivorous species, feeding on discarded fauna may not translate into higher TL if most discarded species have the same TL as their natural prey. Contributions of other, possibly allochthonous food sources (e.g. freshwater or terrestrial subsidies), or variable trophic discrimination factor (TDF) linked to feeding on dead and possibly decomposed animal tissues, might also play a role. For these reasons, we argue that TL assessment alone may not allow to ascertain discard consumption and should be complemented with further diet assessment methods.

Potential discard consumption based on diet reconstruction using stable isotope mixing models was assessed in 10 taxa for which discard consumption was potentially important based on gut content metabarcoding data from the study area [38]. These taxa showed relatively high TL compared to literature values and fitted well within the polygon of sources (i.e. 2 Chondrichthyes, 2 cephalopods, 4 decapods, 1 gastropod and 1 polychaete). However, the fact that food sources were grouped in relatively large trophic guilds that may comprise both discarded and non-discarded taxa due to high isotopic similarity and the fact that identity and frequency of discards may change according to multiple factors (e.g. geographic location or time) [14, 64] generally prevented drawing a clear trophic link between consumers and discards. Contribution of potential discards to the diet of selected taxa could be assessed by pinpointing inconsistencies in predator-prey interactions revealed by mixing model analysis (e.g. feeding on prey that have higher TL or are too large to be predated upon alive, or prey that do not occupy the same habitat). Consumption of pelagic secondary consumers (PSC), a group consisting of Clupeidae and Scombridae, and demersal fish and cephalopods (DFC), a trophic guild regrouping some of the highest TL prey (i.e. tertiary consumers, TL > 4), likely corresponded to discard consumption for all taxa. Indeed, all invertebrates are unlikely to be able to catch and feed upon live fish belonging to these 2 groups except for cephalopods, and they regrouped some of the most discarded species in the area [36, 41]. The small-spotted catshark *S*. *canicula* and the two cephalopods (*Alloteuthis spp*. and *S*. *officinalis*) were the ones with the highest contributions from both the highest TL prey (i.e. demersal fish and cephalopods [DFC]) and pelagic secondary consumers (PSC). The relatively large spread of posterior distributions may reflect population variability linked to generalist and opportunistic feeding behaviors. *S*. *canicula* is known to make a significant use of fisheries discards, playing a role in this species distribution pattern [65]. In another study [22], *S*. *canicula* was also shown to be one of the main discard consumers, they strongly respond to discarding and consume discarded Clupeidae. The natural diet of *Raja undulata* is largely dominated by crustaceans [66], which contrasts with the substantial contributions from pelagic fish (PSC) and demersal fish and cephalopods (DFC) revealed here by mixing model analysis. Scavenging on discards was also noted for Rajidae [22] and cephalopods [18].

Although *Aphrodita aculeata* and *Buccinum undatum* are known to be necrophageous, their diet does not typically include fish prey [62, 67]. Similarly, sampled crabs and spider crabs species are known to feed mainly on invertebrates, and only occasionally on carrion or small fish [62, 68, 69]. Yet here, their cumulated mean contribution could represent up to 30% of the assimilated diet for *C*. *pagurus* (i.e. without considering the fish fraction potentially included in SBF and BIF contributions). Potential contribution of discards could not easily be assessed on the basis of ‘SBF’ and ‘BIF’ proportions in consumers’ diet because both trophic guilds regroup at least some invertebrates or fish species that may be naturally predated upon by all of the studied consumers. Yet, this does not exclude that contributions from these two groups might also correspond to discard consumption to some extent. Potential discard consumption may therefore be underassessed in this regard. Our results are congruent with previous studies recording discard consumption by whelk *B*. *undatum* [19, 70], crabs, hermit crabs and spider crabs [17, 19, 71] and with gut content metabarcoding of a bentho-demersal community in the Bay of Bourgneuf, revealing potentially important discard ingestion in all of these taxa [38]. Decapods constitute the dominant invertebrate Order attracted by or consuming discards, based on a review of 43 observational studies [18]. Most decapods are opportunistic omnivores and the spatial variability in prey availability is the primary determinant of their trophic strategies [68, 69]. Therefore, they may rely more on discards where discarding practices are more important, as suggested in this study.

### Advancing research on discard consumption by integrating multiple diet assessment techniques

Stable isotopes have undeniable strengths such as representing the assimilated diet, as opposed to the ingested diet, being time and space integrative, and allowing the modelling of key trophic attributes such as the trophic level [28–30]. As such, they were useful to model TL and trace potential influence of discard consumption on the isotopic composition of multiple marine species in the Bay of Bourgneuf, mainly benthic invertebrates. However, isotopic similarity between different species and their grouping in large trophic groups via clustering analysis implied low taxonomic resolution of stable isotope mixing models. This prevented us from fully assessing potential discard assimilation and exploring the diversity of food items potentially ingested as discards. This issue illustrates that stable isotopes alone may not allow apprehending the complexity of trophic interactions with discards and the effect of discard consumption on the food web. The repeated use of diet assessment methods providing high resolution in prey items identification, such as DNA metabarcoding of gut contents, might allow clarifying these aspects [38]. Diet information on discard consumption might also be used as informative priors to improve performance of stable isotope mixing models in a Bayesian framework [58]. However, caution should be adopted regarding the fact that informative priors used in stable isotope mixing models should reflect the ‘true’ diet, and integrate diet information over a similar period of time as stable isotopes, otherwise it might introduce biases and actually reduce model performances [72]. It is also worth noting that spatial and temporal changes in δ^15^N values of baselines may also bias the calculation of TL and the interpretation of food web structure if not properly taken into account. Trawling itself, may resuspend nutrients in the water column and affect the isotopic composition of primary producers or consumers [26]. In shallow coastal areas, freshwater input is also an important factor of baseline isotope variation, and by extension, that of the whole trophic chain [46]. For these reasons, we advise to avoid interpretations of results based solely on the analysis of raw δ^15^N values, and to design sampling to include baselines from the entire sampled area in order to be able to convert raw δ^15^N values to TL. Here, we aimed to account for such effects by choosing a restricted area of study that is not subject to important river input [73, 74], with a relative depth homogeneity (5–22 m) and by sampling baselines across the area. Finally, underwater video analysis remains a powerful tool and possibly the only technique allowing to document direct evidence of discard consumption by marine fauna. The integration of all techniques might allow to better interpret stable isotope analyses to assess the importance of the different processes that may potentially affect the TL of marine fauna in fishing areas.

### Forecasting the consequences of discard reductions

Discarding practices constitute a cause of concern for the perennation of marine resources [14] and the European Union recently implemented discard reduction policies [24]. However, predicting the effects of reducing fisheries discards constitute a challenge because we currently lack fine scale assessment of discard consumption at local scale and time-integrative data on trophic links with discards, to evaluate whether it may be widespread across communities, particularly in areas that are subject to important discarding activity [9, 18]. While discards did not constitute main food resources for any of the sampled taxa, our results suggest that they may still constitute substantial portions of the diet of benthic invertebrate macrofauna and Chondrichthyes in the Bay of Bourgneuf. This suggests that these taxa might be more affected by discard reductions. Because decapods usually play an important structuring role within communities, any effect on their population might have indirect consequences affecting the community or habitat structure [69, 75], including in the case of discard reduction [38]. However, this largely depends upon their ability to switch diet and the availability of other food sources. The current study constitutes an assessment of trophic levels, species diet and food web structure before the full implementation of the European Landing Obligation (aiming to reduce discards) in the Bay of Bourgneuf [40]. Future follow up studies may allow to test hypotheses relative to TL changes and dietary switch following discard reductions developed here. Understanding fisheries discards reintegration in marine food webs is crucial in the context of an ecosystem approach to fisheries management, to better understand the functioning of marine ecosystems subject to fishing and anticipate any potential impacts of discard reductions.

## Supporting information

S1 Table Raw stable isotope data. (PDF)

S1 Fig Dendrogram of the clustering analysis using the UPGMA method, highlighting the 6 trophic groups identified within the consumers community. PPC = Pelagic primary consumers (light blue). PSC = Pelagic secondary consumers (blue). BIF = Benthic invertebrates and fish (black). SBF = Shrimps and benthic fish (grey). DFC = Demersal fish and cephalopods (red).
(PDF)
