# Supplementary material for: Assessing the diet and trophic level of marine fauna in a fishing ground subject to discarding activity using stable isotopes
Source: PLoS One. 2022 Jun 7;17(6):e0268758. doi: 10.1371/journal.pone.0268758 (PMC9173610; doi:10.1371/journal.pone.0268758)
Supplement: S1 Table — (PDF) [file pone.0268758.s001.pdf]

S1 Table. Raw stable isotope data.

Group	Genus-species	$\delta^{13}C$ (‰)	$\delta^{15}N$ (‰)	Total length (cm)
Actinopterygii	*C.lyra*	-17.26	13.49	12
Actinopterygii	*C.lyra*	-17.45	13.13	12
Actinopterygii	*C.lyra*	-17.09	12.11	15
Actinopterygii	*C.lyra*	-17.73	12.84	11
Actinopterygii	*C.lyra*	-17.31	12.27	20
Actinopterygii	*C.lyra*	-17.33	11.88	18
Actinopterygii	*C.lyra*	-17.68	12.62	21
Actinopterygii	*C.lucerna*	-15.21	15.50	23
Actinopterygii	*C.lucerna*	-15.35	15.64	23
Actinopterygii	*C.lucerna*	-14.88	15.59	24
Actinopterygii	*C.lucerna*	-15.61	14.81	21
Actinopterygii	*C.lucerna*	-17.61	15.05	23
Actinopterygii	*C.lucerna*	-15.42	15.04	21
Actinopterygii	*C.lucerna*	-15.89	15.16	26
Actinopterygii	*C.conger*	-15.87	16.36	150
Actinopterygii	*C.conger*	-16.62	14.81	114
Actinopterygii	*C.conger*	-16.79	16.05	123
Actinopterygii	*C.conger*	-17.30	15.23	80
Actinopterygii	*C.conger*	-17.05	14.72	76
Actinopterygii	*C.conger*	-16.94	14.78	75
Actinopterygii	*E.encrasicolus*	-18.28	13.63	13
Actinopterygii	*E.encrasicolus*	-18.12	13.62	13
Actinopterygii	*E.encrasicolus*	-18.41	13.13	12
Actinopterygii	*E.encrasicolus*	-17.89	12.09	13
Actinopterygii	*E.encrasicolus*	-18.36	12.29	14
Actinopterygii	*E.encrasicolus*	-17.98	12.79	14
Actinopterygii	*E.encrasicolus*	-18.17	13.34	13
Actinopterygii	*S.pilchardus*	-18.24	11.89	21
Actinopterygii	*S.pilchardus*	-19.43	12.33	11
Actinopterygii	*S.pilchardus*	-18.00	12.39	21
Actinopterygii	*S.pilchardus*	-18.39	13.01	21
Actinopterygii	*S.pilchardus*	-17.68	13.23	19
Actinopterygii	*S.pilchardus*	-17.98	13.85	17
Actinopterygii	*S.sprattus*	-19.32	12.58	7
Actinopterygii	*S.sprattus*	-19.26	12.43	8
Actinopterygii	*S.sprattus*	-19.13	12.35	8
Actinopterygii	*S.sprattus*	-19.49	12.06	9
Actinopterygii	*S.sprattus*	-19.47	11.41	9
Actinopterygii	*S.sprattus*	-19.55	11.69	8
Actinopterygii	*S.sprattus*	-18.59	11.56	11
Actinopterygii	*S.scombrus*	-17.92	13.44	22

Actinopterygii	*S.scombrus*	-18.15	12.50	23
Actinopterygii	*S.scombrus*	-18.78	11.95	22
Actinopterygii	*S.scombrus*	-19.27	11.44	21
Actinopterygii	*S.scombrus*	-18.58	12.44	19
Actinopterygii	*S.scombrus*	-18.24	11.27	22
Actinopterygii	*S.scombrus*	-18.78	11.46	22
Actinopterygii	*O.eperlanus*	-16.20	16.34	13
Actinopterygii	*O.eperlanus*	-17.87	15.49	13
Actinopterygii	*O.eperlanus*	-17.88	15.16	12
Actinopterygii	*O.eperlanus*	-17.84	15.35	12
Actinopterygii	*O.eperlanus*	-17.61	15.83	13
Actinopterygii	*O.eperlanus*	-17.16	14.98	12
Actinopterygii	*O.eperlanus*	-17.33	15.43	13
Actinopterygii	*B.belone*	-18.16	13.69	71
Actinopterygii	*B.belone*	-16.80	15.34	61
Actinopterygii	*B.belone*	-17.47	12.09	71
Actinopterygii	*B.belone*	-17.20	12.31	70
Actinopterygii	*T.trachurus*	-18.21	15.50	28
Actinopterygii	*T.trachurus*	-18.36	15.57	25
Actinopterygii	*T.trachurus*	-17.58	15.13	26
Actinopterygii	*T.trachurus*	-18.22	13.94	26
Actinopterygii	*T.trachurus*	-18.35	15.40	24
Actinopterygii	*T.trachurus*	-18.48	14.62	25
Actinopterygii	*T.trachurus*	-17.54	14.99	27
Actinopterygii	*P.pollachius*	-18.62	14.35	27
Actinopterygii	*P.pollachius*	-18.13	14.47	42
Actinopterygii	*P.pollachius*	-15.50	15.29	23
Actinopterygii	*P.pollachius*	-16.98	15.92	27
Actinopterygii	*P.pollachius*	-17.45	14.87	41
Actinopterygii	*P.pollachius*	-16.97	15.47	41
Actinopterygii	*P.pollachius*	-17.05	15.78	24
Actinopterygii	*T.luscus*	-16.21	16.68	15
Actinopterygii	*T.luscus*	-16.30	16.84	17
Actinopterygii	*T.luscus*	-15.88	15.90	21
Actinopterygii	*T.luscus*	-17.50	14.58	16
Actinopterygii	*T.luscus*	-16.43	15.88	20
Actinopterygii	*T.luscus*	-17.31	15.49	17
Actinopterygii	*T.luscus*	-17.32	14.60	16
Actinopterygii	*M.merlangus*	-17.37	16.72	24
Actinopterygii	*M.merlangus*	-17.87	15.76	18
Actinopterygii	*M.merlangus*	-17.71	16.34	24
Actinopterygii	*M.merlangus*	-17.36	16.72	19
Actinopterygii	*M.merlangus*	-17.16	17.10	26
Actinopterygii	*M.merlangus*	-17.43	17.33	29
Actinopterygii	*M.merluccius*	-18.16	14.32	25

Actinopterygii	*M.merluccius*	-18.06	14.59	25
Actinopterygii	*M.merluccius*	-18.53	13.17	23
Actinopterygii	*M.merluccius*	-17.49	15.09	31
Actinopterygii	*M.merluccius*	-17.66	15.01	28
Actinopterygii	*M.merluccius*	-17.77	14.96	26
Actinopterygii	*M.merluccius*	-18.41	14.68	20
Actinopterygii	*P.pagrus*	-17.18	15.23	21
Actinopterygii	*P.pagrus*	-16.09	16.42	22
Actinopterygii	*P.pagrus*	-16.82	15.73	22
Actinopterygii	*P.pagrus*	-16.41	15.06	21
Actinopterygii	*P.pagrus*	-17.22	16.04	20
Actinopterygii	*P.pagrus*	-16.05	16.06	22
Actinopterygii	*P.pagrus*	-16.66	16.17	21
Actinopterygii	*S.cantharus*	-16.78	15.50	19
Actinopterygii	*S.cantharus*	-17.49	15.57	16
Actinopterygii	*S.cantharus*	-15.93	16.23	18
Actinopterygii	*S.cantharus*	-17.67	15.41	19
Actinopterygii	*S.cantharus*	-17.35	14.69	19
Actinopterygii	*S.cantharus*	-16.78	14.70	20
Actinopterygii	*S.cantharus*	-17.03	15.27	21
Actinopterygii	*L.bergylta*	-18.30	13.63	16
Actinopterygii	*L.bergylta*	-18.24	13.26	14
Actinopterygii	*L.bergylta*	-16.22	13.71	17
Actinopterygii	*L.bergylta*	-16.85	14.57	14
Actinopterygii	*S.solea*	-17.13	13.79	22
Actinopterygii	*S.solea*	-16.25	14.24	23
Actinopterygii	*S.solea*	-16.87	14.12	23
Actinopterygii	*S.solea*	-17.26	13.56	21
Actinopterygii	*S.solea*	-16.42	13.95	25
Actinopterygii	*S.solea*	-20.25	14.39	24
Actinopterygii	*S.solea*	-16.32	14.67	25
Chondrichthyes	*R.undulata*	-16.07	14.81	65
Chondrichthyes	*R.undulata*	-15.42	14.65	57
Chondrichthyes	*R.undulata*	-16.35	14.51	55
Chondrichthyes	*R.undulata*	-15.05	14.48	39
Chondrichthyes	*R.undulata*	-15.93	14.96	48
Chondrichthyes	*R.undulata*	-16.86	13.66	43
Chondrichthyes	*R.undulata*	-16.41	14.79	63
Chondrichthyes	*S.canicula*	-16.56	14.74	57
Chondrichthyes	*S.canicula*	-17.15	14.02	63
Chondrichthyes	*S.canicula*	-16.44	15.15	58
Chondrichthyes	*S.canicula*	-16.54	14.88	50
Chondrichthyes	*S.canicula*	-16.76	14.59	49
Chondrichthyes	*S.canicula*	-16.98	14.53	58
Chondrichthyes	*S.canicula*	-17.28	14.39	50

Cephalopoda	*Alloteuthis.spp*	-18.67	14.51	7
Cephalopoda	*Alloteuthis.spp*	-18.68	13.84	8
Cephalopoda	*Alloteuthis.spp*	-18.92	13.91	7
Cephalopoda	*Alloteuthis.spp*	-19.11	13.50	7
Cephalopoda	*Alloteuthis.spp*	-17.98	17.02	9
Cephalopoda	*Alloteuthis.spp*	-18.05	15.60	7
Cephalopoda	*Alloteuthis.spp*	-19.01	13.96	8
Cephalopoda	*S.officinalis*	-17.08	14.29	11
Cephalopoda	*S.officinalis*	-17.63	14.33	12
Cephalopoda	*S.officinalis*	-17.38	14.81	14
Cephalopoda	*S.officinalis*	-17.41	14.59	12
Cephalopoda	*S.officinalis*	-17.27	15.40	9
Cephalopoda	*S.officinalis*	-17.19	14.39	7
Cephalopoda	*S.officinalis*	-17.17	14.80	10
Decapoda	*A.undecimdentatus*	-16.20	12.26	5
Decapoda	*A.undecimdentatus*	-17.93	12.88	5
Decapoda	*A.undecimdentatus*	-16.40	12.05	7
Decapoda	*A.undecimdentatus*	-16.87	12.04	6
Decapoda	*A.undecimdentatus*	-17.28	12.04	6
Decapoda	*A.undecimdentatus*	-17.34	12.92	6
Decapoda	*A.undecimdentatus*	-17.96	12.40	6
Decapoda	*A.undecimdentatus*	-16.07	13.25	6
Decapoda	*A.undecimdentatus*	-17.90	12.83	4
Decapoda	*C.pagurus*	-16.83	13.72	15
Decapoda	*C.pagurus*	-17.42	13.35	11
Decapoda	*C.pagurus*	-16.53	15.11	15
Decapoda	*C.pagurus*	-17.30	13.27	11
Decapoda	*C.pagurus*	-17.85	13.81	12
Decapoda	*C.pagurus*	-17.13	14.53	10
Decapoda	*C.pagurus*	-17.93	13.49	9
Decapoda	*N.puber*	-17.08	13.42	6
Decapoda	*N.puber*	-17.37	14.04	6
Decapoda	*N.puber*	-16.61	12.63	5
Decapoda	*N.puber*	-16.80	12.36	6
Decapoda	*N.puber*	-16.78	12.33	6
Decapoda	*N.puber*	-16.71	13.04	7
Decapoda	*N.puber*	-16.68	12.75	7
Decapoda	*M.brachydactyla*	-17.09	13.73	13
Decapoda	*M.brachydactyla*	-16.37	13.47	12
Decapoda	*M.brachydactyla*	-16.31	13.60	12
Decapoda	*M.brachydactyla*	-17.06	11.78	11
Decapoda	*M.brachydactyla*	-16.86	13.82	11
Decapoda	*M.brachydactyla*	-16.88	13.07	12
Decapoda	*M.brachydactyla*	-17.35	13.66	14
Decapoda	*Pagurus.spp*	-15.40	12.62	NA

Decapoda	*Pagurus.spp*	-16.97	12.45	NA
Decapoda	*Pagurus.spp*	-16.39	12.86	NA
Decapoda	*Pagurus.spp*	-15.99	13.07	NA
Decapoda	*Pagurus.spp*	-16.29	13.03	NA
Decapoda	*Pagurus.spp*	-16.24	13.08	NA
Decapoda	*Pagurus.spp*	-16.11	13.27	NA
Decapoda	*Crangon.spp*	-15.71	13.91	NA
Decapoda	*Crangon.spp*	-15.05	13.65	NA
Decapoda	*Crangon.spp*	-13.47	13.68	NA
Decapoda	*Crangon.spp*	-16.63	14.10	NA
Decapoda	*Crangon.spp*	-15.83	13.67	NA
Decapoda	*Crangon.spp*	-14.02	14.07	NA
Decapoda	*Crangon.spp*	-16.29	14.12	NA
Polychaeta	*A.aculeata*	-17.15	13.55	8
Polychaeta	*A.aculeata*	-16.71	14.42	6
Polychaeta	*A.aculeata*	-16.49	14.57	6
Polychaeta	*A.aculeata*	-17.55	12.75	8
Polychaeta	*A.aculeata*	-16.30	14.00	8
Polychaeta	*A.aculeata*	-15.49	13.02	7
Gastropoda	*B.undatum*	-16.22	12.92	7
Gastropoda	*B.undatum*	-15.84	13.09	6
Gastropoda	*B.undatum*	-17.12	12.76	7
Gastropoda	*B.undatum*	-17.04	13.78	7
Gastropoda	*B.undatum*	-16.33	13.84	8
Gastropoda	*B.undatum*	-16.04	14.52	7
Gastropoda	*B.undatum*	-16.11	12.93	7
Bivalvia	*P.maximus*	-17.92	10.38	12
Bivalvia	*P.maximus*	-17.88	9.89	12
Bivalvia	*P.maximus*	-18.14	9.72	12
Bivalvia	*P.maximus*	-17.75	10.17	12
Bivalvia	*P.maximus*	-17.75	9.85	13
Bivalvia	*P.maximus*	-18.12	9.75	12
Bivalvia	*P.maximus*	-17.94	10.79	11
