# Supplementary figures and images for: Assessing the diet and trophic level of marine fauna in a fishing ground subject to discarding activity using stable isotopes
Source: PLoS One. 2022 Jun 7;17(6):e0268758. doi: 10.1371/journal.pone.0268758 (PMC9173610; doi:10.1371/journal.pone.0268758)

UPGMA clustering dendrogram

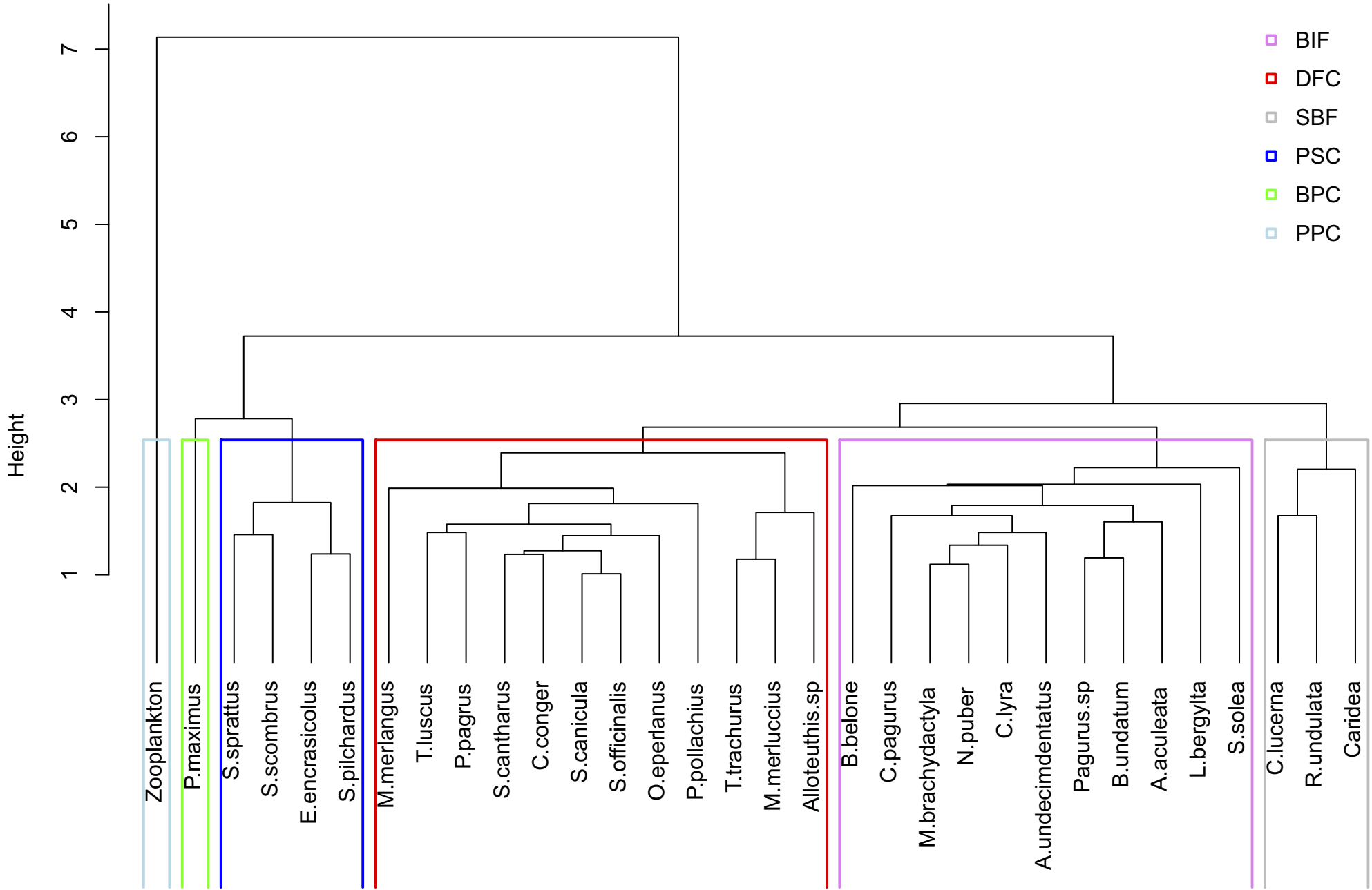

32 taxa  
6 groups

Supplement: S1 Fig — PPC = Pelagic primary consumers (light blue). PSC = Pelagic secondary consumers (blue). BIF = Benthic invertebrates and fish (black). SBF = Shrimps and benthic fish (grey). DFC = Demersal fish and cephalopods (red). (PDF) [file pone.0268758.s002.pdf]
